# Chlorogenic acid in green bean coffee on body weight: a systematic review and meta-analysis of randomized controlled trials

**DOI:** 10.1186/s13643-023-02311-4

**Published:** 2023-09-14

**Authors:** Sukrit Kanchanasurakit, Surasak Saokaew, Pochamana Phisalprapa, Acharaporn Duangjai

**Affiliations:** 1 Division of Clinical Pharmacy, Department of Pharmaceutical Care, School of Pharmaceutical Sciences, University of Phayao, Phayao, 56000 Thailand; 2 Division of Pharmaceutical Care, Department of Pharmacy, Phrae Hospital, Phrae, 54000 Thailand; 3 Center of Health Outcomes Research and Therapeutic Safety (Cohorts), School of Pharmaceutical Sciences, University of Phayao, Phayao, 56000 Thailand; 4 Unit of Excellence On Clinical Outcomes Research and IntegratioN (UNICORN), School of Pharmaceutical Sciences, University of Phayao, Phayao, 56000 Thailand; 5 Unit of Excellence On Herbal Medicine, School of Pharmaceutical Sciences, University of Phayao, Phayao, 56000 Thailand; 6 Division of Social and Administration Pharmacy, Department of Pharmaceutical Care, School of Pharmaceutical Sciences, University of Phayao, Phayao, 56000 Thailand; 7 Division of Ambulatory Medicine, Department of Medicine, Faculty of Medicine Siriraj Hospital, Mahidol University, Bangkok, 10700 Thailand; 8 Unit of Excellence in Research and Product Development of Coffee, Division of Physiology, School of Medical Sciences, University of Phayao, Phayao, 56000 Thailand

**Keywords:** Green bean coffee, Chlorogenic acid, Body weight, Obesity, Meta-analysis

## Abstract

**Background:**

Supplemental green bean coffee extract (GBCE) with caffeine has been shown to prevent weight gain. There are different dosages of GBCE that contain chlorogenic acid (CGA), and the data for their effectiveness in preventing weight gain (500 mg/day) is currently out of date. To better understand the effects of GBCE containing CGA on body weight, the present study sets out to perform a systematic review and meta-analysis of these studies.

**Methods:**

Using electronic databases, including Scopus, Embase, PubMed, and Cochrane Library databases, literature was searched up to October 13, 2022. For the meta-analysis examining the impact of GBCE containing CGA (500 mg/day) on body weight with a random-effects model, the randomized controlled trials (RCTs) were considered. We calculated weighted mean differences and 95% confidence intervals (CIs). To gauge study heterogeneity, the Cochran Q statistic and I-squared tests (*I*^2^) were employed.

**Results:**

The meta-analysis includes three RCTs with 103 individuals (case = 51, control = 52). The combined findings of GBCE with CGA at least 500 mg/day result in body weight reduction (*WMD*: − 1.30 and 95% *CI*: − 2.07 to − 0.52, *p* = 0.001) without study heterogeneity (*I*^2^ = 0%, *p* = 0.904) and without publication bias estimated using Egger’s and Begger’s test (*p* = 0.752 and *p* = 0.602, respectively).

**Conclusions:**

According to the meta-analysis, GBCE with CGA 500 mg/day lowers body weight. Nevertheless, despite its limited sample size and short-term study, this study was successful. Long-term research on the effectiveness and safety of GBCE and CGA on body weight require more clinical trials.

**Systematic review registration:**

PROSPERO CRD42021254916.

**Supplementary Information:**

The online version contains supplementary material available at 10.1186/s13643-023-02311-4.

## Background

Type 2 diabetes, hypertension, cardiovascular illness, musculoskeletal disorders, Alzheimer’s disease, depression, and cancer have all been linked to obesity as a global health concern [1]. Obesity is defined as having an excessive amount of body weight. A body mass index (BMI) over 25 kg/m^2^  is indicated as overweight, while over 30 kg/m^2^ is considered as obese [2]. Several strategies were recommended to manage overweight and obesity including dietary, lifestyle, physical activity, behavior modification, and surgery [2]. Additionally, prescription drugs such orlistat, lorcaserin, liraglutide, naltrexone/bupropion, and phentermine/topiramate were considered for long-term therapy. However, adverse drug reactions, such as nausea, vomiting, hypoglycemia, disorientation, and diarrhea, as well as stomach discomfort, constipation, and diarrhea, are common [3]. Due to their effectiveness and safety in managing obesity, alternative medicines, particularly natural botanicals, seem to be receiving greater attention. Weight loss vomiting has been induced by *Cissus* *quadrangularis (CQ)*, *Sambucus nigra*, *Asparagus officinalis*, *Garcinia atroviridis*, *Garcinia cambogia*, green tea, caffeine, nephedrine, capsaicin, yohimbine, chitosan, and guar gum [4, 5].

Because green bean coffee (GBC) contains potent nutrients and bioactive substances including caffeine, caffeic acid, and chlorogenic acids (CGA), it is currently believed to help with weight loss. Many clinical trials supported that GBC is associated with reducing the risk of insulin resistance, obesity, [6, 7] and anti-inflammatory and antioxidant properties [8] and has been found to be safe for consumption [9]. Besides, CGA exhibits antidiabetic, [10] anti-lipidemic, and anti-obesity properties [11] since their regulated glucose and lipid metabolism and inhibited lipid absorption [10, 12]. CGA and caffeic acid were suggested to improve body weight, lipid metabolism, and obesity-related hormone levels in high-fat diet-induced mice, which may through changing plasma adipokine level and body fat distribution and suppressing the activities of fatty acid synthase, 3-hydroxy-3-methylglutaryl coenzyme-A reductase (HMGCR), and acyl-CoA:cholesterol acyltransferase (ACAT), whereas stimulating fatty acid β-oxidation activity and peroxisome proliferator-activated receptors (PPARα) expression in the liver [11]. Additionally, CGA diminish body weight and fat deposition which may be related to peroxisome proliferator-activated receptor gamma, coactivator 1α (PGC-1α), and uncoupling Protein 1 (UCP1) in the monosodium glutamate (MSG)-induced obesity mouse model and the oleic acid-induced HepG2 cells [13]. Amano et al. (2019) supported that CGA was safe and advantageous in pharmaceuticals [14].

According to a new thorough review and dose–response meta-analysis of randomized controlled trials, green coffee extract decreases obesity by reducing body weight and BMI [15]. The study is quite heterogeneous, and there are several GBCE dosages that contain CGA. The current evidence of the GBCE covering CGA supplementation (more than or equal to 500 mg/day) against weight gain, however, is not well updated. Therefore, the goal of the systematic review and meta-analysis of randomized controlled trials was to answer the following research question: Does receiving GBCE containing at least 500 mg of CGA per day (intervention) compared to placebo (comparator) affects on body weight (outcome) in participants either healthy or metabolic disease (population)?

## Method

### Protocol and registration

This systematic review and meta-analysis was carried out and reported in accordance with the Preferred Reporting Items for Systematic Reviews and Meta-Analyses (PRISMA) statement. This study was documented in PROSPERO (registration number: CRD42021254916). Since information was gathered and aggregated from earlier research, the systematic review and meta-analysis was exempt from ethical approval, and neither patients nor the public was engaged in this investigation. The patient data has been deidentified and made available to the public.

### Data sources and search strategy

The Scopus, Embase, PubMed, and Cochrane Library databases were thoroughly searched from their establishment until October 13, 2022. The Medical Subject Headings (MeSH) were utilized when necessary. We looked through the bibliographies of relevant papers. The following keywords were used in the search approach, with minor database-based adjustments: “body weight,” “body mass index,” and “body fat,” as well as “coffee,” “caffeine,” “green bean coffee,” and “chlorogenic acid.” There were no restrictions on language.

### Study selection

We considered the randomized controlled trials (RCTs) studies that (I) involved patients over the age of 18; (II) provided the data for main outcome, i.e., body weight that can be calculated as mean, standard deviation (SD), for data pooling; (III) had a comparison group that either received a placebo or did not receive green coffee extract; and (IV) looked at the impact of green coffee extract on body weight. In addition to being excluded were studies involving animals and those that were not presented as original research, reviews, observational studies, comments, editorials, expert opinions, surveys, letters, abstracts from conference meetings, case reports, case series, systematic reviews, and meta-analyses. Studies using a dosage of chlorogenic acid less than 500 mg were also rejected. Studies involving the same individuals were also disregarded if the researchers failed to provide impact estimates or if there was not enough information to calculate effect estimates.

### Data extraction and quality assessment

Two researchers individually examined each title, abstract, and full-text publication for possibly appropriate studies (S. K. and A. D.). Any disagreements were discussed between two researchers and a third to find a solution (S. S.). Data were taken from all articles that could have been connected by the same team. The following information was taken from each study: authors, published year, study region, study design, participant characteristics, sample size, study length, intervention and comparator details, and outcome measurement (A. D.). We contacted the appropriate authors when there was a shortage of outcome data. If the author did not react within a month, the study was deemed invalid. All the obtained data was independently assessed by two researchers (S. S. and A. D.). The main focus of assessment was the change in body weight observed during an 8-week period following the commencement of treatment, encompassing both participants with a healthy condition and those diagnosed with a metabolic disease.

Two investigators, S. K. and A. D., independently evaluated the quality of individual studies using the Cochrane risk-of-bias (RoB) tool version 2.0 for randomized trials and the RoB 2.0 for crossover trials, as applicable. The assessment encompassed several criteria, including bias arising from the randomization process, bias arising from period and carryover effects (for crossover trials), bias due to deviations from intended intervention, bias due to missing outcome data, bias in measurement of the outcome, and bias in selection of the reported result. In the event of disagreements, discussions were held to resolve.

### Data synthesis and statistical analysis

DerSimonian-Laird random-effects models were used to quantify pooled effects for the study and explain the relationship between consumption of green bean coffee and body weight using the weighted mean difference (WMD) and 95% confidence interval (CI). In case of the crossover trial, we employed paired analysis in accordance with the methodology recommended by Elbourne et al. [16] The Q statistic developed by Cochran was used to measure heterogeneity. An alpha value of 0.10 was selected for each analysis to show trial heterogeneity. *I*^*2*^ values showed how heterogeneous the data was. *I*^2^ values more than 75%, 25–75%, and less than 25%, in that order, indicate significant, moderate, and low heterogeneity, respectively. When there existed heterogeneity, an effort was made to investigate its possible causes. To measure publication bias, the funnel plot, Egger’s test, and Begg’s test were utilized.

### Quality of evidence

The Grading of Recommendations, Assessment, Development and Evaluation (GRADE) approach was used to rate the quality of evidence of estimates. Using the GRADEpro GDT software online version (https://www.gradepro.org/), the risk of bias, inconsistency, indirectness, imprecision, and publication bias were used to assess the quality of the evidence for the result. Four categories of evidence levels may be made: high, moderate, low, and extremely low.

## Result

### Study selection

**Fig. 1:**
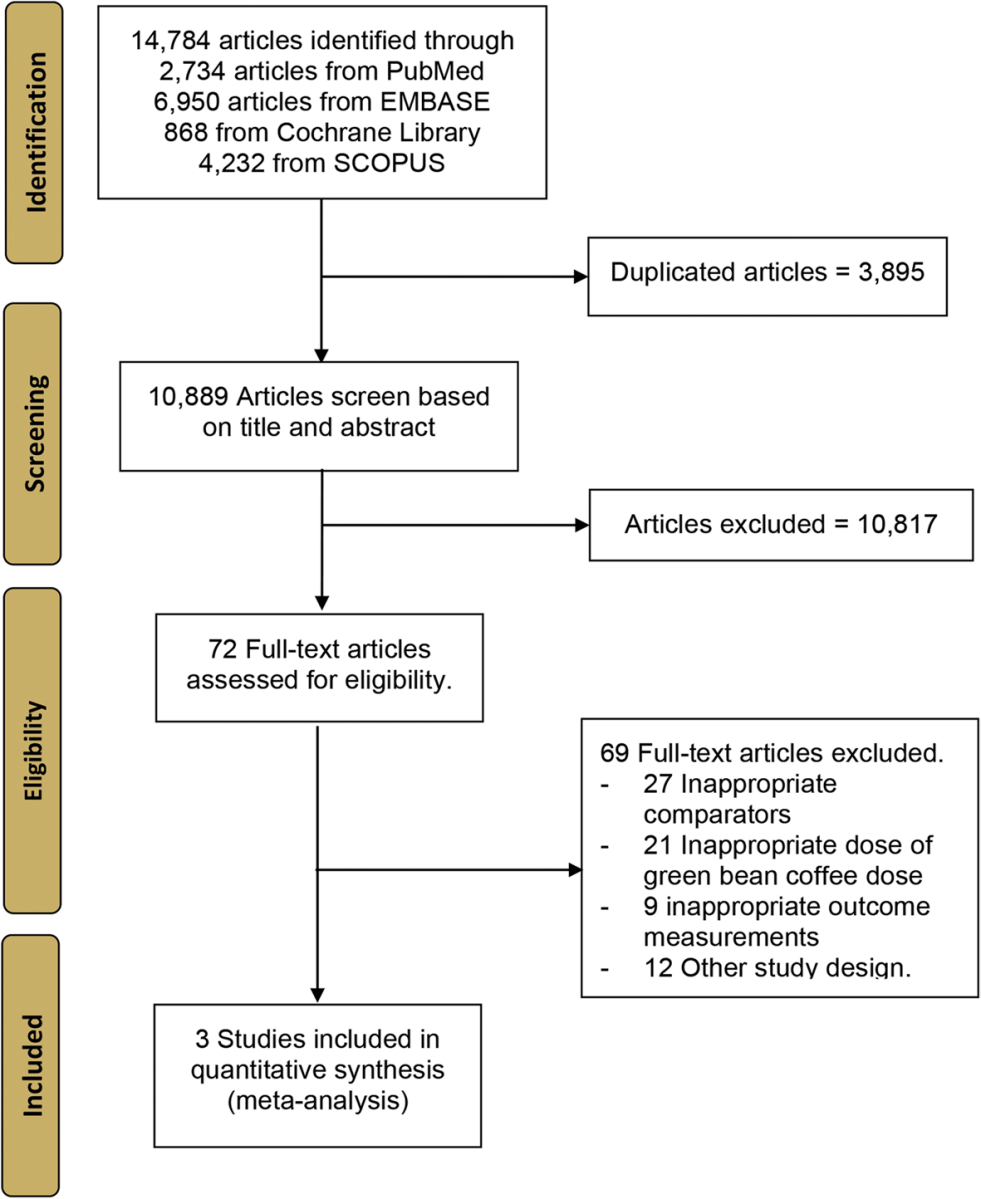
PRISMA flow diagram

Our first literature search using Scopus, Embase, PubMed, and Cochrane Library databases from conception to October 13, 2022, yielded a total of 16,017 items. A total of 11,829 publications were filtered based on the title and abstract; 82 full-text papers were then read and evaluated for eligibility. In the end, a total of three full-text articles were incorporated into the synthesis of this systematic review and meta-analysis. Figure 1 illustrates a flow diagram for the Preferred Reporting Items for Systematic Reviews and Meta-Analyses. Supplementary Table S1 gives all the specifics of our literature search.

### Characteristics of the included studies

The characteristics of the included studies are summarized in Table 1. Between 2016 and 2018, these articles appeared in publications. The impact of green coffee beans on body weight was assessed in every study. In addition to one research in the UK, two studies were carried out in Iran. A sample size ranging from 16 to 44 individuals was used for the overall meta-analysis, which involved 110 persons (51 case and 52 control). Participants were 39.96 ± 12.67 years old on average (aged between 18 and 70 years). One to 8 weeks were spent in therapy across trials.

**Table 1:** Characteristics of the included studies

Author	Year	Region of study	Study design	Age (year)^a^	Male/ female	Number of participants	Detail of intervention and comparator	Outcome measurement^a^	Timepoint for outcome measurement
Al-Dujaili E. A. S	2016	Jordan	Crossover placebo-controlled trial	24.6 ± 3.3	7/9	16	Intervention Chlorogenic acid 500 mg per day Comparator • Placebo	Body weight (kg) Intervention group • 72.16 ± 16.53 (at day 0) • 71.64 ± 16.35 (1 week later) Comparator group • 72.16 ± 16.53 (at day 0) • 72.10 ± 16.45 (1 week later)	1 week after baseline
Shahmohammadi H. A	2017	Iran	Randomized controlled trial	42.93 ± 6.5	22/22	44	Intervention • Green coffee bean extract capsule 500 mg (contain 250 mg of chlorogenic acid) take 1 capsule before breakfast and lunch Comparator • Placebo	Body weight (kg) Intervention group • 88.81 ± 6.73 (at day 0) • 85.68 ± 5.73 (8 weeks later) Comparator group • 90.25 ± 6.99 (at day 0) • 88.60 ± 6.69 (8 weeks later)	8 weeks after baseline
Roshan H	2018	Iran	Randomized controlled trial	52.36 ± 9.3	10/33	43	Intervention • Green coffee bean extract 400 mg twice per day (800 mg per day) Comparator • Placebo	Body weight (kg) Intervention group • 78.10 ± 11.01 (at day 0) • 76.01 ± 10.52 (8 weeks later) Comparator group • 80.11 ± 12.45 (at day 0) • 79.18 ± 12.75 (8 weeks later)	8 weeks after baseline

^a^Mean (± SD). *mg* milligram, *kg* kilogram

### Effect of green bean coffee on body weight

**Fig. 2:**
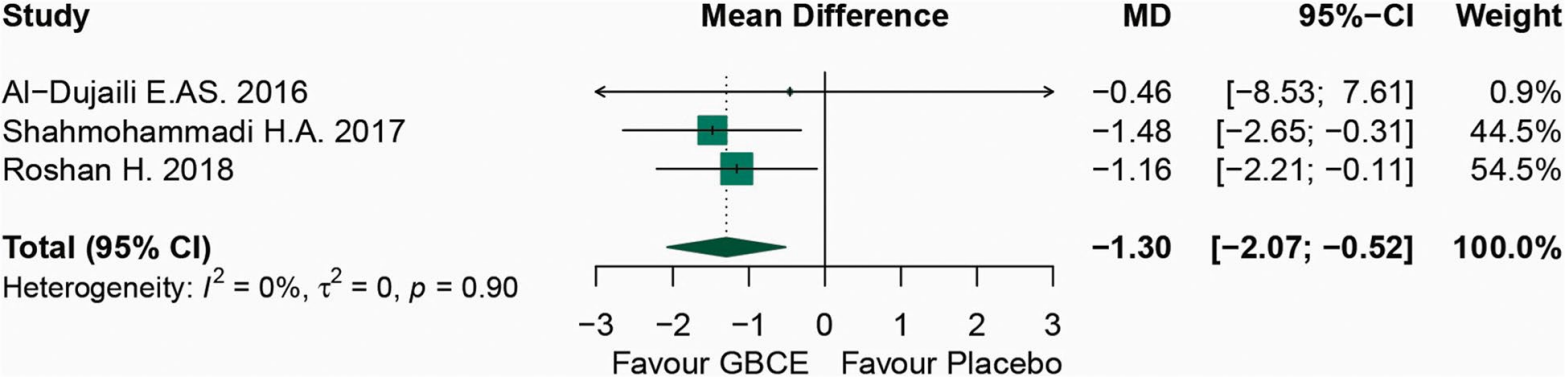
Forest plot of randomized controlled trials considering the effects of green bean coffee extract (GBCE) on body weight

In Fig. 2, the forest plots for the effect of a green coffee bean supplement on body weight are displayed. It presented the combined body weight data between the GBCE and placebo groups of three investigations. In general, the random effects model demonstrated that GBCE supplements reduced body weight when compared to taking a placebo (weight mean difference: WMD): − 1.30 and 95% *CI*: − 2.07 to − 0.52, *p* = 0.001). The studies have been shown no evidence of heterogeneity (*I*^2^ = 0%, *p* = 0.904). These findings indicated that GCBE (500 mg/day) may have decreased body weight by 1.30 kg.

### Assessment of quality and risk of bias

**Fig. 3:**
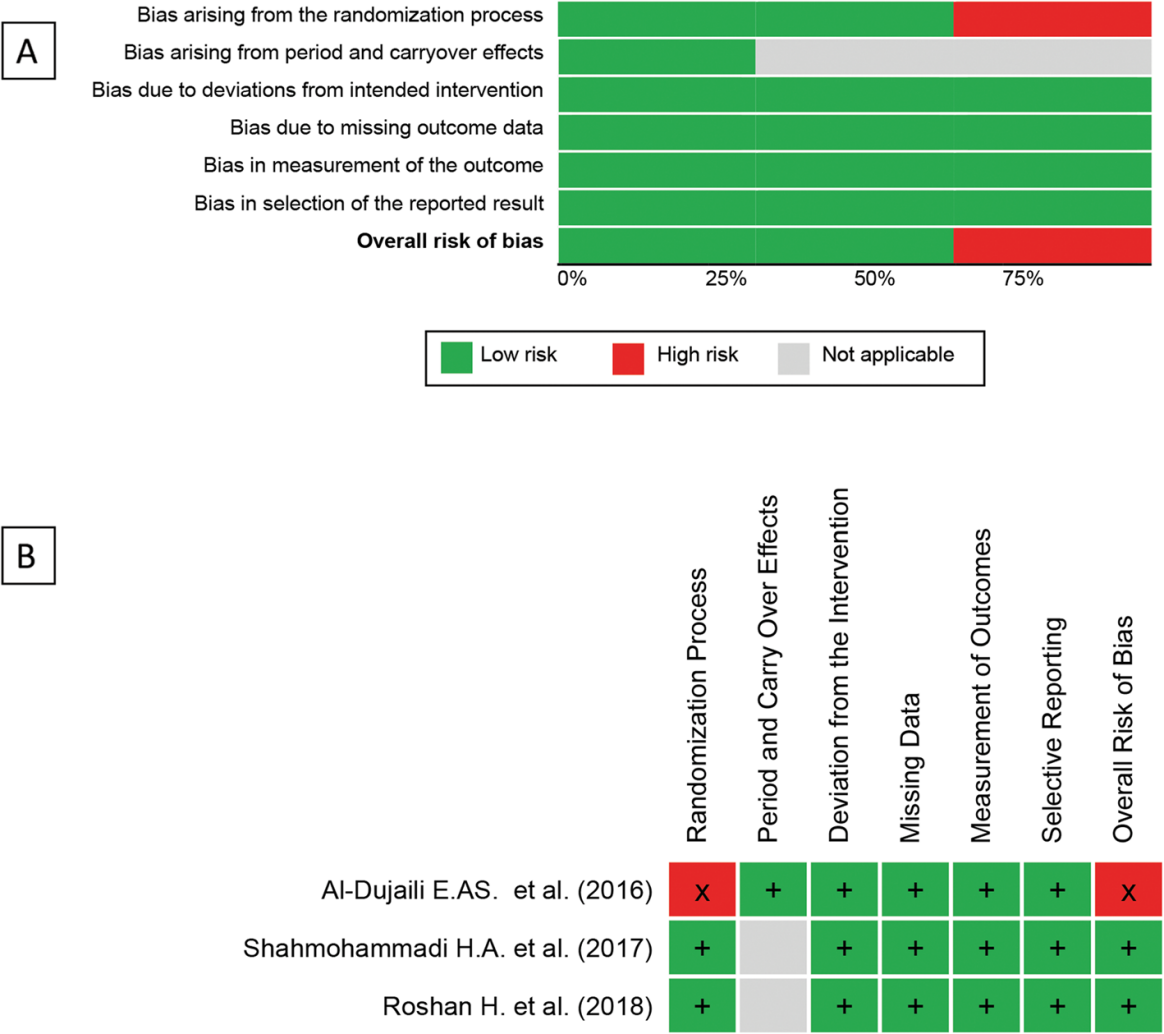
Quality of bias assessment of the included studies according to the Cochrane risk-of-bias tool (RoB 2.0) and the RoB 2.0 for crossover trials. Green, low risk of bias; yellow, some concerns; red, high risk of bias; gray, not applicable (not a crossover trial)

The Cochrane criteria were used to evaluate the study’s quality and risk of bias, as shown in Fig. 3. The risk of bias was high in one study and low in other studies. Begg’s and Egger’s tests were employed to detect any potential publication bias among the studies we included in our meta-analyses. Using Egger’s and Begger’s regression tests for (*p* = 0.752 and *p* = 0.602, respectively), no statistically significant publication was discovered.

### Quality of evidence

Table 2 displays the level of evidence provided by the RCT included in the meta-analysis. High grades on the GRADE system are given to randomized trials without substantial constraints. High risk of bias was assigned to one of the included RCT studies. As a result, we decided to lower the rating for the bias risk domain. The intervention and outcome measures were predicted to be comparable among the included RCT trials, in addition to low heterogeneity. Inconsistency and indirectness were not thought to be critical levels of certainty. We opted to lower the rating for imprecision because the CI showed a likely benefit from the intervention and comparison methods.

**Table 2:** The quality of evidence of the randomized controlled trial included for meta-analysis

Outcome	Number of studies	Study design	Quality assessment					Weighted mean difference (95% *CI*)	Quality
			**Risk of bias**	**Inconsistency**	**Indirectness**	**Imprecision**	**Other considerations**		
Body weight	3	RCT	Serious^a^	Not serious^b^	Not serious^c^	Serious^d^	None^e^	− 1.28 (− 2.03; − 0.54)	** ⊕ ⊕ ◯◯** Low

^a^One trial was high risk of bias. ^b^Inconsistency explained by *I*^2^ value as 0%; low heterogeneity. ^c^The intervention and outcome measured were similar in included studies. ^d^The confidence interval includes possible benefit from intervention and comparator approaches. ^e^None: Publication bias is not likely. *RCT* randomized control trial, *CI* confidence interval

## Discussion

The caffeine and other chemical components in green bean coffee come from raw, unroasted coffee beans. According to our research, GBCE with 500 mg/day of CGA reduces body weight.

Multiple comprehensive analyses of clinical studies have demonstrated that GBCE supplementation can encourage weight reduction, lower obesity, fasting blood glucose levels, and improve lipid profiles [6, 17, 18]. Furthermore, GBCE has activity against on fat accumulation [19]. Shimoda et al. (2006) give evidence that green coffee bean extract administration reduces weight gain and visceral fat accumulation which may be involved in their active compounds, caffeine and CGA [19]. Coffee bean extract is considered to reduce adipose tissue weight and to attenuate body weight gain by increasing lipogenic enzyme activity of mitochondrial carnitine palmitoyltransferase (CPT) in the liver and decreasing lipolytic enzyme of cytosolic fatty acid synthetic (FAS), malic enzyme, and glucose 6-phosphate dehydrogenase (G6PDH) activity [20]. Green coffee bean suppresses adipogenesis involved in wingless-type MMTV integration site family 10b (WNT10b) and galanin-mediated adipogenesis cascades by downregulating genes peroxisome proliferator-activated receptor γ2 (PPARγ2) and CCAAT/enhancer-binding protein α (C/EBPα) [21]. It has been reported that CGA termed as 5-caffeoylquinic acid (5-CQA) improved obesity through stimulating the AMP-activated protein kinase (AMPK), inhibits 3-hydroxy-3-methylglutaryl coenzyme-A reductase (HMGCR), and enhanced the activity of carnitine palmitoyl transferase [22]. CGA reduced body weight and fat deposition by possibly involving in PGC-1α and UCP1 in obesity mouse and HepG2 cells [13] and regulating fatty acid β-oxidation by activating PPARα in the liver. [11]

With the minimum effective dose, 0.3% of green coffee bean extract exhibited for regulating body weight gain, fat accumulation, and insulin resistance in mice fed the high-fat diet (HFD) for 11 weeks [21]. Once to convert human dose equivalent based on the normalization to body surface area as proposed by Reagan-Shaw et al. (2007), [23] 0.3% green coffee bean extract in mice relates to approximately 1460 mg/60 kg body weight in human [21], while systematic review and clinical trials reported that green coffee consumption at 200 to1000 mg/day for 1 to 8 weeks reduced body weight [6, 8, 24, 25]. Administration CGA-7 at 500 mg for 12 weeks in humans was confirmed the safety that CGA-7 did not alter biochemical and hematological parameters and the markers of hepatic toxicity and vital sign and had no undesirable effects. However, it has been noted that the compound was treated with a short duration and a smaller sample size [7]. Amano et al. (2019) support the safety pharmacological activities of CGAs and its metabolites in in vitro and ex vivo studies according to the guideline on safety pharmacology studies for human pharmaceuticals (ICH S7A) [14]. They suggested that CGAs have fundamental properties and safe for usage in pharmaceuticals [14].

The current systematic review and meta-analysis addressed the effect of green bean coffee extract (GBCE) containing CGA (≥ 500 mg/day) in the dietary supplement on body weight. The meta-analysis was conducted on 110 people with aged between 18 and 70 years. In all studies, GBCE was administered daily, for between 1 and 8 weeks at a dose of 800 to 1000 mg/day. Tablet administrations of GBCE were found in two studies, while another study was performed as capsules. Our data showed that GBCE containing CGA 500 mg/day reduces body weight 1.28 kg. All the RCTs concerning use of green bean coffee on body weight have been performed with small sample sizes and short duration. Our study revealed a low heterogeneity (0%) that demonstrated the reliability and stability of this meta-analysis. However, there is limited clinical trials evidence of efficacy and safety of GBCE and CGA on body weight in long-term studies. Onakpoya et al. (2010) showed the efficacy of green coffee extract (GCE) decreases weight loss with three RCTs; sample size in the trials ranged 30 to 62 [18]. The meta-analysis revealed green coffee extract (GCE) at 180 to 200 mg/day containing 40 to 45% of CGA for 4 to12 weeks to reduce body weight 2.47 kg (*MD*: − 2.47 kg; 95% *CI*: − 4.23, − 0.72) [18]. All studies exhibit a high risk of bias and have moderate magnitude of the effect size and represent heterogeneity 97% [18]. Additionally, two RCTs of CGA-enriched GCE found no statistically significant difference to reduce body weight between GCE and placebo (*MD*: − 1.92 kg; 95% *CI*: − 5.40, 1.56) with heterogeneity 99% [17]. Any of the clinical trials reported adverse effects of GCE supplement [18]. Following GCE intake, physiological parameters (heart rate, energy intake, or sodium intake) remained unchanged [18]. After the trial, the lipid profile parameters (serum TAG, TC, LDL cholesterol, and HDL-cholesterol concentrations), glycated hemoglobin (HbA1c), and physical activity (metabolic equivalent of task) did not alter [6]. No adverse (AE) or serious adverse events (SAEs) of vital signs, serum biochemical indicators of liver function, or hematological results have been seen while taking CGA-7 at 500 mg for 12 weeks [7]. Reportedly, involved in the capacity to scavenge reactive oxygen species are phenolic chemicals found in abundance in green coffee beans [26]. Other studies conducted the meta-analysis study from 15 articles with GCE at 46 to 6000 mg/day containing CGA about 28–54% for 1 to 12 weeks; sample size ranged 20 to 70 [15]. GCE supplement no significant change in body weight (*WMD*: − 0.585 kg, 95% *CI*: − 1.498, 0.329,* p* = 0.210) with heterogeneity among the studies (*I*^*2*^ = 92.4%, *p* < 0.001) [15]. Hausenblas and Huynh (2014) displayed green coffee bean extract supplementation to reduce weight loss from 6 RCTs, the extract at 46 to 1050 mg/day for 4 to 12 weeks [27]. The extract supplement contains CGA about 40–54% reducing weight (*MES* = 0.55, 95% *CI*: 0.05, 1.05, *P* = 0.03) with heterogeneity among the studies *I*^*2*^ = 83.24 [27]. Based on analysis and mentioned above, the current study indicated that the existence of CGA in GBCE may benefit on body weight diminishing. Nevertheless, a suitable formulation of supplement, doses, and timing of administration are more important factors for monitoring weight control.

To examine the therapeutic effectiveness of GBCE on body weight, it should be emphasized that our meta-analysis study contains several strengths and limitations. The key advantage is that a comparable sample of the same size and a specific GBCE source are provided, whereas the limitations include the difference of population study in healthy volunteers, [24] patients with the metabolic syndrome [6], and patients with nonalcoholic fatty liver disease [8]. However, there was not much statistical heterogeneity. Additionally, there are several varied demographics, short treatment durations among trials, and variable GBCE administration dosage forms. To determine the validity of suggestions, however, extensive research with large samples is required.

## Conclusion

GBCE was discovered to be a trend to lower body weight in this comprehensive review and meta-analysis of RCTs. There is a need for more prospective research with bigger sample sizes.

### Supplementary Information

**Additional file 1: Table S1.** Search algorithms.

## Data Availability

The dataset supporting the conclusions of this article is included within the article and its Supplementary file 1.

## Abbreviations

ACAT: Acyl-CoA:cholesterol acyltransferase
AMPK: Adenosine monophosphate-activated protein kinase
BMI: Body mass index
C/EBPα: CCAAT/enhancer-binding protein α
CIs: Confidence intervals
CGA: Chlorogenic acid
CPT: Carnitine palmitoyltransferase
*CQ*: *Cissus* *quadrangularis*
5-CQA: 5-Caffeoylquinic acid
FAS: Fatty acid synthetic
GBC: Green bean coffee
GBCE: Green bean coffee extract
G6PDH: Glucose 6-phosphate dehydrogenase
GRADE: The Grading of Recommendations, Assessment, Development and Evaluation
*I*^2^: I-squared tests
HFD: High-fat diet
HMGCR: 3-Hydroxy-3-methylglutaryl coenzyme-A reductase
MeSH: The Medical Subject Headings
MSG: Monosodium glutamate
PGC-1α: Peroxisome proliferator-activated receptor gamma coactivator 1α
PRISMA: The Preferred Reporting Items for Systematic Reviews and Meta-Analyses
PPARα: Peroxisome proliferator-activated receptors
RCTs: Randomized controlled trials
RoB: Risk of bias
SD: Standard deviation
UCP1: Uncoupling protein 1
WMD: Weight mean difference
WNT10b: Wingless-type MMTV integration site family 10b
PPARγ2: Peroxisome proliferator-activated receptor γ2

## References

[1] Blüher M (2019). Obesity: global epidemiology and pathogenesis. Nat Rev Endocrinol.

[2] Gadde KM, Martin CK, Berthoud H-R, Heymsfield SB. Obesity: pathophysiology and management. J Am Coll Cardiol. 2018;71(1):69–84. 10.1016/j.jacc.2017.11.011 PMC7958889 29301630

[3] Erlandson M, Ivey LC, Seikel K (2016). Update on office-based strategies for the management of obesity. Am Fam Physician.

[4] Hasani-Ranjbar S, Nayebi N, Larijani B, Abdollahi M (2009). A systematic review of the efficacy and safety of herbal medicines used in the treatment of obesity. World J Gastroenterol: WJG.

[5] Bahmani M, Eftekhari Z, Saki K, Fazeli-Moghadam E, Jelodari M, Rafieian-Kopaei M (2016). Obesity phytotherapy: review of native herbs used in traditional medicine for obesity. J Evid Based Complement Altern Med.

[6] Roshan H, Nikpayam O, Sedaghat M, Sohrab G (2018). Effects of green coffee extract supplementation on anthropometric indices, glycaemic control, blood pressure, lipid profile, insulin resistance and appetite in patients with the metabolic syndrome: a randomised clinical trial. Br J Nutr.

[7] Sudeep H, Shyam PK (2021). Supplementation of green coffee bean extract in healthy overweight subjects increases lean mass/fat mass ratio: a randomized, double-blind clinical study. SAGE Open Medicine.

[8] Shahmohammadi HA, Hosseini SA, Hajiani E, Malehi AS, Alipour M. Effects of green coffee bean extract supplementation on patients with non-alcoholic fatty liver disease: a randomized clinical trial. Hepatitis Monthly. 2017;17(4):e12299.

[9] Bagchi D, Verma N, Mittal M, Swaroop A, Bagchi M, Preuss HG (2017). Safety and efficacy of a novel green coffee bean extract (GCB‐70) in overweight subjects. FASEB J.

[10] Meng S, Cao J, Feng Q, Peng J, Hu Y. Roles of chlorogenic acid on regulating glucose and lipids metabolism: a review. Evid Based Complement Altern Med. 2013;2013:801457. 10.1155/2013/801457 PMC3766985 24062792

[11] Cho A-S, Jeon S-M, Kim M-J, et al. Chlorogenic acid exhibits anti-obesity property and improves lipid metabolism in high-fat diet-induced-obese mice. Food Chem Toxicol. 2010;48(3):937–43. 10.1016/j.fct.2010.01.003 20064576

[12] Naveed M, Hejazi V, Abbas M (2018). Chlorogenic acid (CGA): a pharmacological review and call for further research. Biomed Pharmacother.

[13] Zhong Y, Ding Y, Li L (2020). Effects and mechanism of chlorogenic acid on weight loss. Curr Pharm Biotechnol.

[14] Amano Y, Honda H, Nukada Y (2019). Safety pharmacological evaluation of the coffee component, caffeoylquinic acid, and its metabolites, using ex vivo and in vitro profiling assays. Pharmaceuticals.

[15] Gorji Z, Varkaneh HK, Nazary-Vannani A, et al. The effect of green-coffee extract supplementation on obesity: a systematic review and dose-response meta-analysis of randomized controlled trials. Phytomedicine. 2019;63:153018. 10.1016/j.phymed.2019.153018 31398662

[16] Elbourne DR, Altman DG, Higgins JP, Curtin F, Worthington HV, Vail A (2002). Meta-analyses involving cross-over trials: methodological issues. Int J Epidemiol.

[17] Ding F, Ma B, Nazary-Vannani A (2020). The effects of green coffee bean extract supplementation on lipid profile in humans: a systematic review and meta-analysis of randomized controlled trials. Nutr Metab Cardiovasc Dis.

[18] Onakpoya I, Terry R, Ernst E. The use of green coffee extract as a weight loss supplement: a systematic review and meta-analysis of randomised clinical trials. Gastroenterol Res Pract. 2010;2011:382852. 10.1155/2011/382852 PMC2943088 20871849

[19] Shimoda H, Seki E, Aitani M (2006). Inhibitory effect of green coffee bean extract on fat accumulation and body weight gain in mice. BMC Complement Altern Med.

[20] Tanaka K, Nishizono S, Tamaru S, et al. Anti-obesity and hypotriglyceridemic properties of coffee bean extract in SD rats. Food Sci Technol Res. 2009;15(2):147–52.

[21] Song SJ, Choi S, Park T. Decaffeinated green coffee bean extract attenuates diet-induced obesity and insulin resistance in mice. Evid Based Complement Altern Med. 2014;2014:718379. 10.1155/2014/718379 PMC4003760 24817902

[22] Kumar R, Sharma A, Iqbal MS, Srivastava JK (2020). Therapeutic promises of chlorogenic acid with special emphasis on its anti-obesity property. Curr Mol Pharmacol.

[23] Reagan-Shaw S, Nihal M, Ahmad N (2008). Dose translation from animal to human studies revisited. FASEB J.

[24] Al-Dujaili EA, Abuhajleh MN, Al-Turk W (2016). Effect of green coffee bean extract consumption on blood pressure and anthropometric measures in healthy volunteers: a pilot crossover placebo controlled study. Jordan J Pharm Sci.

[25] Thom E (2007). The effect of chlorogenic acid enriched coffee on glucose absorption in healthy volunteers and its effect on body mass when used long-term in overweight and obese people. J Int Med Res.

[26] Palmieri, Miguel Gontijo Siqueira, et al. Enhancement of antioxidant properties from green coffee as promising ingredient for food and cosmetic industries. Biocatalysis and agricultural biotechnology. Biocatalysis Agri Biotechnol. 2018;16:43–48.

[27] Hausenblas H, Huynh B. Effect of green coffee bean extract on weight loss. Publish Nat Med J*.* 2014;6(3).

