# Supplementary material for: Chlorogenic acid in green bean coffee on body weight: a systematic review and meta-analysis of randomized controlled trials
Source: Syst Rev. 2023 Sep 14;12:163. doi: 10.1186/s13643-023-02311-4 (PMC10503105; doi:10.1186/s13643-023-02311-4)
Supplement: Supplementary file 1 — Additional file 1: Table S1. Search algorithms. [file 13643_2023_2311_MOESM1_ESM.docx]

**Supplementary data**

**Table S1. Search algorithms**

**Table S1. Search algorithms.**

| **Database** | **No** | **Step search algorithm** | **Items found** |
| --- | --- | --- | --- |
| Pubmed | #1 | coffee | 18949 |
|  | #2 | caffeine | 37400 |
|  | #3 | "green bean coffee" | 250 |
|  | #4 | "chlorogenic acid" | 6589 |
|  | #5 | coffee OR caffeine OR "green bean coffee" OR "chlorogenic acid" | 58207 |
|  | #6 | "body weight" | 373387 |
|  | #7 | "body mass index" | 284430 |
|  | #8 | "body fat" | 37836 |
|  | #9 | "body weight" OR "body mass index" OR "body fat" | 638545 |
|  | **#10** | **(coffee OR caffeine OR "green bean coffee" OR "chlorogenic acid") AND ("body weight" OR "body mass index" OR "body fat")** | **2870** |
| Cochran | #1 | coffee | 2065 |
|  | #2 | caffeine | 5292 |
|  | #3 | "green bean coffee" | 59 |
|  | #4 | "chlorogenic acid" | 192 |
|  | #5 | #1 OR #2 OR #3 OR #4 | 6639 |
|  | #6 | "body weight" | 79158 |
|  | #7 | "body mass index" | 53856 |
|  | #8 | "body fat" | 23280 |
|  | #9 | #6 OR #7 OR #8 | 96100 |
|  | **#10** | **#5 AND #9** | **951** |
| EMBASE | #1 | coffee | 59795 |
|  | #2 | caffeine | 115025 |
|  | #3 | "green bean coffee" | 379 |
|  | #4 | "chlorogenic acid" | 11949 |
|  | #5 | #1 OR #2 OR #3 OR #4 | 92772 |
|  | #6 | "body weight" | 813151 |
|  | #7 | "body mass index" | 325775 |
|  | #8 | "body fat" | 69072 |
|  | #9 | #6 OR #7 OR #8 | 1093879 |
|  | **#10** | **#5 AND #9** | **7598** |
| SCOPUS | #1 | coffee | 48370 |
|  | #2 | caffeine | 64783 |
|  | #3 | "green bean coffee" | 18 |
|  | #4 | "chlorogenic acid" | 17218 |
|  | #5 | #1 OR #2 OR #3 OR #4 | 121928 |
|  | #6 | "body weight" | 630163 |
|  | #7 | "body mass index" | 301650 |
|  | #8 | "body fat" | 64344 |
|  | #9 | #6 OR #7 OR #8 | 894511 |
|  | **#10** | **#5 AND #9** | **4598** |
